# Randomised controlled trial of a new palliative care service: Compliance, recruitment and completeness of follow-up

**DOI:** 10.1186/1472-684X-7-7

**Published:** 2008-05-28

**Authors:** Irene J Higginson, Sam Hart, Rachel Burman, Eli Silber, Tariq Saleem, Polly Edmonds

**Affiliations:** 1 King's College London, School of Medicine, Department of Palliative Care, Policy and Rehabilitation, Denmark Hill, London SE5 9RJ, UK; 2 King's College Hospital NHS Trust, Department of Neurology, Denmark Hill, London SE5 9RS, UK

## Abstract

**Background:**

Palliative care has been proposed for progressive non-cancer conditions but there have been few evaluations of service developments. We analysed recruitment, compliance and follow-up data of a fast track (or wait list control) randomised controlled trial of a new palliative care service – a design not previously used to assess palliative care.

**Methods/Design:**

An innovative palliative care service (comprising a consultant in palliative medicine, a clinical nurse specialist, an administrator and a psychosocial worker) was delivered to people severely affected by multiple sclerosis (MS), and their carers, in southeast London. Our design followed the MRC Framework for the Evaluation of Complex Interventions. In phase II we conducted randomised controlled trial, of immediate referral to the service (fast-track) versus a 12-week wait (standard best practice). Main outcome measures were: compliance (the extent the trial protocol was adhered to), recruitment (target 50 patients), attrition and missing data rates; trial outcomes were Palliative Care Outcome Scale and MS Impact Scale.

**Results:**

69 patients were referred, 52 entered the trial (26 randomised to each arm), 5 refused consent and 12 were excluded from the trial for other reasons, usually illness or urgent needs, achieving our target numbers. 25/26 fast track and 21/26 standard best practice patients completed the trial, resulting in 217/225 (96%) of possible interviews completed, 87% of which took place in the patient's home. Main reasons for failure to interview and/or attrition were death or illness. There were three deaths in the standard best practice group and one in the fast-track group during the trial. At baseline there were no differences between groups. Missing data for individual questionnaire items were small (median 0, mean 1–5 items out of 56+ items per interview), not associated with any patient or carer characteristics or with individual questionnaires, but were associated with interviewer.

**Conclusion:**

This is the first time a fast track (or wait list) randomised trial has been reported in palliative care. We found it achieved good recruitment and is a feasible method to evaluate palliative care services when patients are expected to live longer than 3–6 months. Home interviews are needed for a trial of this kind; interviewers need careful recruitment, training and supervision; and there should be careful separation from the clinical service of the control patients to prevent accidental contamination.

**Trial Registration:**

Clinical Trials.Gov NCT00364963

## Background

Trials of new palliative care services are rare and beset with serious methodological problems, in some instances so severe that the studies fail [1]. Systematic reviews have attempted to assess the effectiveness of palliative home care [2], multidisciplinary teams [3,4], day care [5], support for carers [6], services for older people [7] and in dementia [8]. They identified: (1) a wealth of studies concerned with need rather than treatment, and (2) problems of trial compliance, recruitment, attrition, contamination, bias, outcome measurement and definition of the intervention.

This has led some to question whether randomised controlled trials are appropriate in palliative care [1,8]. In addition, ethical concerns have been raised about randomising patients who may be near the end of life [9]. However, non-randomised and quasi-experimental studies suffered from similar problems to those found in trials [4-6]. Robust evaluation and trial methods are urgently needed. Modelled on clinical trials, with phase I, II and III studies, the MRC framework for the evaluation of complex interventions has recently provided a useful model for developing and evaluating services or other complex treatments [10,11]. The recommendation for palliative care to expand to encompass non-cancer patients and cancer patients earlier in their illness urgently needs evaluation. New methods are required to test services in this context.

Multiple sclerosis (MS) is one condition where palliative care is felt to be needed. This chronic disease of the central nervous system affects over 2.5 million people worldwide and is the most common cause of neurological disability in adults under 60 years [12]. As the disease affects many parts of the central nervous system it may be accompanied by marked physical and psychosocial symptoms. In a small proportion of patients the disease may result in profound disability leading to a need for the provision of complex care and, for some people, information on planning around end of life care. Although for many the prognosis is good, around 15% of those affected have primary progressive disease from the outset and a further 35% develop a progressive course after many years of relapsing illness. Thus palliative care may have a role for patients with complex problems, particularly for those with progressive disease.

We designed and sought to determine whether a new palliative care service for people severely affected by MS improves their outcomes. However, because poor recruitment, attrition and missing data are commonly high in palliative care studies [2-4] we wished to test a design not previously used in palliative care – a fast track (also called wait list) trial. This analysis aimed to assess the results of recruitment, compliance, and follow-up of this trial design in this complex group of patients. The design sought to minimise missing data for any reason; and when it was present to understand its patterns and influencing factors. Therefore, we tested the following null hypotheses: 1) missing data would not be associated with patient disability; 2) missing data would not be associated with particular measurement instruments. We explored whether missing data was associated with any other patient, outcome or study characteristics, to inform future imputations. We believe that lessons from our recruitment and trial compliance will add to knowledge in the field and further the development and application of appropriate research in palliative care.

## Methods

### Design, Project Advisory Committee, Ethical (IRB) approval

**Figure 1:**
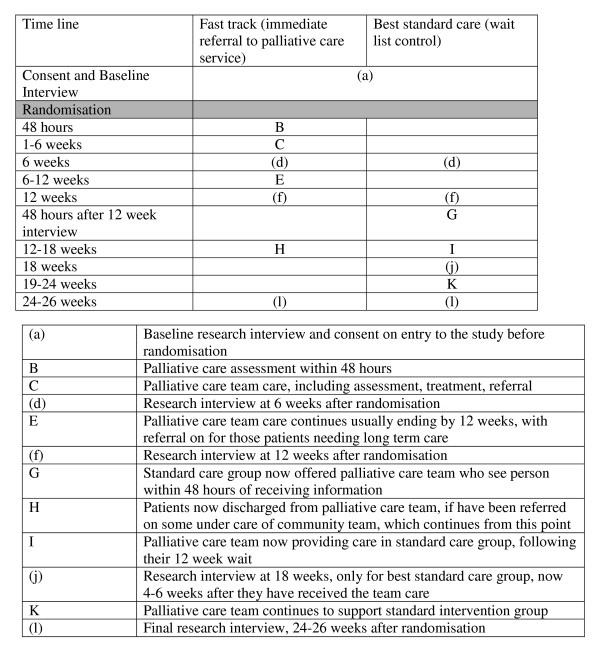
Graphical depiction of intervention in fast track versus standard care trial of a palliative care service.

We followed the Medical Research Council (MRC) Framework for the Evaluation of Complex Interventions [10,11]. This follows a similar framework to that of clinical trials and comprises 5 phases: pre-clinical (theory) and phases I (service modelling), II (preliminary evaluation of service), III (full randomised controlled trial), IV (wide adoption and effectiveness studies). We have previously reported the pre-clinical and phase I findings, which comprised qualitative studies [13-15] and systematic literature reviews [16]. Here we report phase II – an open randomised controlled trial to test subsequent evaluation methods and a preliminary comparison of the new service with standard best practice. Figure 1 uses a graphical method [17] to depict the interventions, interviews and timings in the trial.

Although not all phase II MRC Framework studies comprise a randomised trial, we wished to do so because: (1) to begin to operate the service without a trial would make it very difficult to introduce a trial subsequently, and might lead to ethical concerns among staff because of an 'apparent' reduction in service, making a phase III trial impossible, and (2) we felt it important to test the subsequent trial methods. However, we chose to conduct a fast-track versus standard care intervention trial (also called a wait list trial) because we were concerned about contamination and poor uptake in the control group – a problem found in many other randomised controlled trials of palliative care services [3,4]. In the fast track (wait list) design all patients have the possibility to receive the service, some immediately, and others after a wait (equivalent to a normal wait for NHS services). The detailed protocol is presented elsewhere [13] (registered at clinicaltrials.gov, NCT00364936). A multidisciplinary Project Advisory Committee (PAC) was established for the duration of the project. Research ethics committee approval from King's College Hospital local research ethics committee was granted for all phases of the project.

#### Patients and carers

Inclusion criteria were: Patients in South East London who were living with MS and were deemed (by staff – MS nurses, neurologists, rehabilitation staff, primary care staff, social workers – and in a few instances via voluntary groups and self referrals) – to have specialist palliative care needs. These referral guidelines were circulated in advance and explained in an accompanying education programme and were defined as problems or unresolved issues with: symptom control, psychosocial needs, end of life, advanced planning (directive and competency or consent), planning needs for or difficulty with nutrition and/or hydration or a complex combination of problems. A consultant in palliative medicine (PE or IJH) not involved in delivering the service screened all referrals. Patients were excluded if the referring staff and the screening deemed they had very urgent needs or were deteriorating rapidly. In this instance immediate referral to the service was offered, and the patient was withdrawn from the trial (required for 5/69 patients). Clinical information was recorded where possible on those excluded from the evaluation to allow further understanding of clinical issues [13].

#### Randomisation

Consenting patients were randomised after the baseline interview to two arms. One arm received input from the palliative care team immediately (fast track) in addition to standard best practice services. The other arm was offered the palliative care team only after a > 3 month wait, and until then received only best practice (standard best practice) (Figure 1). Statistical colleagues, independent of the research and clinical team, registered the patients, conducted the randomisation and informed the research team, who then informed the patients, and if patients were "fast track" passed their details to the clinical team. The randomisation used the minimisation method [18] to give an equal balance between groups of the following: gender, age, date of diagnosis, and whether patients could or could not communicate.

#### Standard best practice

Local services, which were available to all those who received the new palliative care service immediately and after a delay, included: MS nurse specialists, district nurses, social services, general practitioners and hospital neurology services. A few patients received home physiotherapy, occupational therapy and/or attended specialist rehabilitation services or clinics and/or other specialist involvement, including continence advice, psychiatry and/or psychology. After 12 weeks of care, patients randomised to this group were offered referral to the palliative care team, as for the fast track group, as if they had been on a waiting list. We chose 12 weeks because the average waiting time for a neurology outpatient appointment at the time was 12–16 weeks, and because we felt that the team was likely to demonstrate an effect in this time. Our pre-clinical and phase I research and advice from the treating neurologist had suggested that most (if not all) patients would still be alive at 12 weeks, making such a wait a realistic option for them.

#### Fast-track (palliative care team)

This comprised a part-time palliative medicine consultant (who had received training in both palliative medicine and neurology), a palliative care nurse (working 3–4 days per week on average), a psychosocial worker (shared with the hospital palliative care team, who was on maternity leave for 7 months during the project), and a service co-ordinator/administrator. This structure was agreed during our modelling phase based on patient and caregiver needs [13-15]. Following referral a team member would aim to visit a referral to make a comprehensive palliative care assessment within 48 hours (see Figure 1). Following assessment by the team, patients who had not seen a neurologist for some time or requiring review by neurologist were referred to a specialist clinic run by a neurologist with an interest in MS (ES). MS nurses, neurologists, social workers and other staff involved in the care of patients, made referrals. Service met regularly with neurologists and MS nurses to discuss patients of concern. The service aimed to complement and not to replace existing services. Patients were visited in their own homes or in some instances hospital outpatient clinics, nursing homes or hospital. After assessment the team suggested ways to improve management of physical, emotional, social and other problems, provided specialist welfare benefits advice and bereavement support, liaised with and acted as a catalyst with local services, both primary and specialist teams. Follow-up occurred as required, depending on clinical need. The service modelling phase, and a pilot service run by the consultant (RB) suggested that a short term assessment and care was required, with symptom control and other actions occurring during 1–3 visits and then referral on the community palliative care teams if longer follow-up was needed. The team recorded whether they assessed patients to have specialist palliative care needs, as in the referral criteria, and the number of assessments, follow-up and subsequent referrals made.

#### Research interviews

Face to face interviews were conducted according to a standardised schedule, using trained interviewers, all with previous experience interviewing in palliative care. The interviewers read the questionnaires and outcome measures to the patients, showing them large cards of the possible response categories for standard scales. Interviewers recorded their responses. After the baseline interview, interviews were repeated for both groups at 6 weeks, 12 weeks, and 24–26 weeks (final interview). In addition, at 18 weeks the standard practice group were interviewed (aiming to have an interview 6 weeks after receipt of the palliative care service) (see Figure 1). Because of the high number of interviews needed at certain times of the study, and because of staff changes, 10 different interviewers were used at stages of the project, but one member of staff conducted the majority of interviews. Questionnaires were selected following a systematic literature review and piloting [16], and in addition to the outcome measures (see below) assessed clinical and demographic circumstances and cognitive function (Abbreviated Mental Test Score, AMTS).

#### Primary outcome measure

For this analysis our main outcomes were trial compliance (the extent the trial protocol was adhered to), recruitment (percentage included in the trial), attrition (percentage lost to follow-up) and missing data. As our main outcome point was at 12 weeks (before cross over) we also report how may interviews were conducted at this point. To assess patient outcomes we used self-reported questionnaires using the Palliative Care Outcome Scale (POS) [19,20] and a specially adapted symptom version, POS-MS symptoms [21] as measured 12 weeks. The former included 8 items on anxiety, patient and carer concerns, practical needs and the latter 18 questions specifically relating to MS symptoms, both using a 0–4 scale.

#### Secondary outcome measures

Self-reported quality of life and impact of MS using the Multiple Sclerosis Impact Scale (MSIS) [22,23] which comprises 29 questions (rated 1–5). We also recorded the patient and caregiver reported use of health and social services (and experience of hospital services) in order to calculate the costs of services, to determine whether the team supplemented or substituted existing services and together with the outcome data to calculate cost effectiveness.

In addition, to determine the patients functional status we used the United Kingdom Neurological Disability Scale – UNDS [24], which comprises 12 sections to assess disability – and using the Expanded Disability Status Scale – EDSS [25] a single item 10 point interviewer assessed scale, a the first and final interviews. We recorded the time taken for the whole interviews.

#### Carer measures

Carers/families self completed a short separate questionnaire assessing carer burden (using the short form Zarit Carer Burden Inventory, 12 items each rated 0–4) and mastery (using the Lawton caregiver mastery scale, 4 items rated 0–4); either during the patient interview but separately, or returned the questionnaire by post.

#### Amendments to procedures

Two amendments were made (following further ethical committee approval) early in the study, after reviewing recruitment of the first 5–6 patients.

(1) Initially patients refusing to enter the trial were offered the fast track service immediately. This was changed so that patients refusing the study, and without immediate/pressing clinical needs, were offered standard best practice.

(2) We lowered our threshold for including patients with cognitive and communication difficulties. We always sought to include all patients where consent and an interview were possible (using technological aids and longer interviews where appropriate). Initially those with cognitive impairment and severe difficulties communicating were excluded because of questions about their ability to provide informed consent. However, as cognitive dysfunction may be a feature of severe MS, we felt that it was important to include this group of patients in the evaluation. After discussion with the PAC it was agreed that patients with severe cognitive impairment were offered inclusion in the trial, provided their carers/families were also in agreement. It was felt that being offered a potentially new service that might help them would be in their best interests and that excluding such patients would mean excluding some individuals which future palliative care services might specifically target.

#### Analysis

In this analysis we determined the response rate, reasons for acceptance and trial refusal, attrition, follow-up rate and pattern of missing data and we explored whether these were related to any patient or study factors. To test our null hypotheses we tested for correlations between the amount of missing data and levels of patient disability using Spearman's rho, and compared the level of missing data for the different measurement instruments and interviewers using one way ANOVA. In addition, we tested whether there were differences between randomisation groups at baseline.

#### Sample Size

We had estimated that a sample of 25 patients in each arm would enable us to detect differences of >1.6 on the POS (for individual items) at p < 0.05, power 80%. The main time point for analysis of the main trial was 12 weeks. Large studies have shown that patients with an EDSS of 8 (defined as being unable to walk with limited use of the upper limbs) or more make up about 15% of the MS population [26]. Based on the local patient numbers of people with an EDSS of ≥ 8 we estimated we would identify 3–4 patients per week, and with 60–70% uptake would recruit and follow up 2 of these. Recruitment over 1 year would therefore give us 50–52 patients, to give an indication whether differences between groups were emerging.

## Results

### Initial recruitment and randomisation

A total of 69 patients were referred to the trial over 11 months. Of these 17 were excluded from the trial. Reasons for trial exclusion were: deemed to have urgent need (symptoms and/or deterioration) and so received fast track service immediately (5); refused consent for the trial (5); had communication difficulties too severe to include (3, note 2 of these occurred in the early stages before we broadened the inclusion criteria); lived outside the visiting area (for service and interviewers) (1); did not have MS (1); language or cognitive problems (1) and administrative error (1) when the family member contacted the team directly, and was mistakenly not offered the trial but instead received the service immediately.

Of the 5 who refused consent, in one instance this was by the family and four instances by the patient. Two refusals (one patient and one family member) were in the early stages of recruitment before the protocol was amended; therefore these two patients were offered and accepted the fast-track service. One family member stated that they did not want their relative to 'risk waiting three months' because they 'have had to wait for lots of things'. After the protocol was amended the refusal rate declined. The other three refusals declined both the study and the service.

The remaining 52 patients were randomised to receive fast track palliative care (26) or standard best practice (26). Thus our planned sample size was achieved in one year of recruitment. Table 1 shows the characteristics of all patients, those randomised to each arm and excluded from the study. The characteristics of all groups were similar: over 90% were white, the age ranged 33–75 and two thirds were women. The majority (96%) had either secondary progressive MS or primary progressive MS. Other disease types included relapsing remitting MS and similar inflammatory CNS diseases such as (Neuromyelitis optica). The mean disease duration was 18 years (SD 9.6 years) before referral to our study; range 0 (a recent diagnosis) to 55 years. On average patients had a high degree of disability, with a mean EDSS score 7.8 (Table 1). Eleven out of 51 patients had scores of 9 or more meaning that they were confined to bed. The EDSS and other measures between the two groups were similar at baseline.

**Table 1:** Characteristics of patients in the two randomised arms and excluded from the study at baseline (n = 69)

	**Fast Track (FI) (n = 26)**		**Standard Practice (SI) (n = 26)**		**Excluded from study (n = 17)**	
**Gender**						
Male	9	*35%*	7	*27%*	4	*24%*
Female	17	*65%*	19	*73%*	13	*76%*
**Age**						
Mean	52.9	--	53.0	--	53.7	--
Median	53.0	--	51.5	--	53.0	--
SD	10.5	--	10.4	--	12.2	--
**Ethnic Group (grouped)**						
White (UK, Irish, European)	23	*88%*	24	*92%*	13	*93%*
Any other group	3	*12%*	2	*8%*	1	*7%*
Not known					3	
**Type of MS**						
Primary Progressive MS	13	*50%*	10	*38%*	5	*33%*
Secondary Progressive MS	12	*46%*	14	*54%*	7	*47%*
Other (inc Relapsing Remitting and Devics)	1	*4%*	2	*8%*	3	*20%*
Not known					2	
**Education: continue after min school leaving age?**						
Yes	11	*42%*	14	*54%*	--	--
No	15	*58%*	12	*46%*	--	--
**Education: degree or equivalent qualification?**						
Yes	9	*35%*	6	*23%*	--	--
No	17	*65%*	20	*77%*	--	--
**Employment status**						
Retired	6	*23%*	5	*19%*	--	--
In employment or self-employed (PT/FT)	0	*0%*	1	*4%*	--	--
Unable to work (due to illness)	20	*77%*	20	*77%*	--	--
**Did the patient ever work in health or social services?**						
Yes	4	*15%*	6	*23%*	--	--
No	22	*85%*	20	*77%*	--	--
**Informal carer**						
No informal carer	6	*23%*	3	*12%*	3	*23%*
Partner (wife/husband)	14	*54%*	15	*58%*	7	*54%*
Offspring (daughter/son)	4	*15%*	3	*12%*	1	*8%*
Sibling (sister/brother)	1	*4%*	2	*8%*	0	*0%*
Parents	1	*4%*	3	*12%*	1	*8%*
All others	0	*0%*	0	*0%*	1	*8%*
**Next of Kin**						
No next of kin	0	*0%*	1	*4%*	0	*0%*
Wife/partner	8	*31%*	3	*12%*	3	*23%*
Husband/partner	7	*27%*	12	*46%*	4	*31%*
Daughter	1	*4%*	3	*12%*	1	*8%*
Son	6	*23%*	0	*0%*	2	*15%*
Brother	1	*4%*	1	*4%*	0	*0%*
Sister	0	*0%*	1	*4%*	1	*8%*
Parents	3	*12%*	5	*19%*	1	*8%*
Any other carer	0	*0%*	0	*0%*	1	*8%*
Not known					4	
**Type of Carer Contact**						
Lives alone	5	*19%*	4	*15%*	3	*25%*
Lives with carer	20	*77%*	21	*81%*	9	*75%*
Other carer contact	1	*4%*	1	*4%*	0	*0%*
Not known					5	
**FUNCTION AND SYMPTOM SCALES AT BASELINE**						
**UNDS TOTAL**						
Mean, (Median)	28.2	(27.5)	29.5	(28.5)	--	--
SD	8.9	--	9.2	--	--	--
N completed	26		26			
**EDSS score**						
Mean	7.7	(8.0)	7.9	(8.0)	--	--
SD	1.0	--	1.0	--	--	--
N completed	25		26			
**MSIS Physical score**						
Mean	67.7	(69.0)	67.0	(70.0)	--	--
SD	18.6	--	10.9	--	--	--
N completed – all items	18		12			
**MSIS Psychological score**						
Mean	20.8	(21.0)	24.6	(25.0)	--	--
SD	7.5	--	10.7	--	--	--
N completed – all items	21		17			
**Core POS total**						
Mean	14.2	(14.0)	13.3	(14.0)	--	--
SD	3.5	--	4.2	--	--	--
N completed – all items	18		15			
**POS MSS total score**						
Mean	18.7	(16.0)	18.4	(18.0)	--	--
SD	9.4	--	10.7	--	--	--
N completed – all items	23		21			
**Zarit Burden Inventory total**						
Mean	20.5	(19.0)	22.5	(23.0)	--	--
SD	10.7	--	6.5	--	--	--
N completed – all items	13		17			

Note: N = 26 for both FI and SI groups, unless indicated for functional and symptom scales. There were no significant differences between any of the groups for any variable, at baseline.

### Trial follow-up and attrition

**Figure 2:**
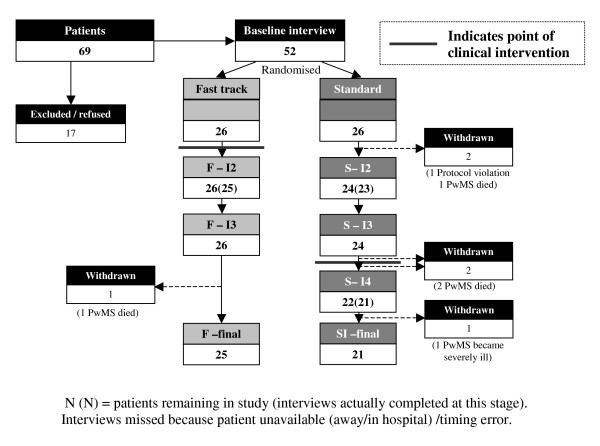
CONSORT DIAGRAM showing flow of patients through the study.

A total of 25/26 fast-track and 21/26 standard best practice patients completed the trial, see Figure 2. One patient initially entered the trial but was excluded because of protocol violation. This patient consented and was randomised to the standard intervention, but due to an administrative error was seen by the service after only a baseline interview, rather than at 12 weeks. Because of limited resources and the preliminary nature of the investigation the principal investigators (PE and IJH) took the decision to continue offering the service, but stop further research data collection, as any such data would have needed to be analysed separately. The patient was fully informed about the ending of research interviews. To ensure that this error did not occur again, subsequently, when patients were randomised to standard best practice, details were kept with the research team until after the third interview at 12 weeks.

Main reasons for loss to follow-up were death or illness. The causes of three deaths in the standard best practice group were: myocardial infarction (2), pneumonia complicated by pyogenic pleural effusion with respiratory failure leading to high dependency admission, complicated by iatrogenic injury in a patient receiving chemotherapy (1). The one patient in the fast-track group who died had spent a great deal of time deteriorating in hospital with recurrent infections and pressure sores, and was subsequently discharged home, as her wish was to die at home. One further patient in the fast-track group has subsequently died, after the end of the study.

By 12 weeks, 26/26 patients had questionnaires returned in the fast track group and 22/26 (and 23/26 for one questionnaire) in the standard track group (with some missing data for individual questionnaire items) (Tables 2 and 3). As Table 4 shows the interviews occurred at similar time intervals in fast track and standard groups and at the times intended (Table 4). At 24–26 weeks, in the fast track group out of a total possible 104 patient interviews, 102 (98%) were completed – one interview did not occur because the patient died and one was missed because of an error in interview timing. In the standard track group there were 115/130 (88%) completed interviews; eight were not possible because patients had died, four because of the protocol violation, and three patients were too unwell to participate in one interview. Of those patients who were alive, we completed 217/225 (96%) possible interviews.

**Table 2:** Amount of missing data for outcome measures in the study, by interview, for patient interviews

**Interview and type **(long or shorter)	***potential n interviews***	**Total n items/questions within interviews**	**N patients (%) with complete data**	**Total n missing items**	**% Missing items**	**Mean (SD) missing items**	**Median missing items**	**Min**	**Max**
**Baseline**	*52*	69	28 (54%)	288	8.0%	5.5 (11.6)	0	0	56
**I2**	*48*	56	35 (72%)	357	12.8%	7.1 (17.5)	0	0	56
**I3**	*50*	56	42 (84%)	103	3.7%	2.1 (9.1)	0	0	56
**I4**	*21(SI only)*	56	18 (86%)	92	3.1%	4.4 (15.3)	0	0	56

**Final**	*47*	69	20 (95%)	69	4.8%	3.3 (15.1)	0	0	69

Note: There were three outliers – a patient who could not complete questionnaire in any interview, three missed interviews – I2 (2) and I4 (1) because of patients being away/in-hospital. These are included in the above analysis. Analysis without these outliers reduces the missing data even further, especially the number of missing items as all questionnaire items were missing for these patients. Open response text fields are not included as these were for additional comments if individuals wished to make them

**Table 3:** Main outcome measures of the study and data completion by study arm and interview

**Questionnaire**	**Multiple Sclerosis Impact Scale (MSIS)**		**Palliative Care Outcome Scale (POS)**		**POS-MS symptoms (POS-MS)**	
Number of items	29		8		18	

Nature of questions	Impact of symptoms on day to day life in last 2 weeks graded 1 (no problem) to 5 (worst problem)		Effect of the problem in the last 3 days graded 0 (no problem) to 4 (overwhelming effect)		Effect of symptom in the last 3 days graded 0 (no problem) to 4 (overwhelming effect)	

**Study arm**	**Fast track**	**Standard**	**Fast track**	**Standard**	**Fast track**	**Standard**

**Interview 1 (baseline)**						
Questionnaires returned/Initial patients entered in study arm (%)	25/26 (96%)	24/26 (92%)	25/26 (96%)	21/26 (81%)	26/26 (100%)	24/26 (92%)
**Reasons for non completion**						
Patient too disabled to self-report*	-	1	-	1	-	1
Questionnaire missed at interview **	1	1	1	4	-	1
N questionnaires with every item completed +	18	12	18	15	23	21
**Interview 2 (4–6 weeks)**						
Questionnaires returned/Initial patients entered in study arm (%)	23/26 (88%)	21/26 (81%)	22/26 (85%)	21/26 (81%)	24/26 (92%)	22/26 (85%)
**Reasons for non completion**						
Patient died	-	1	-	1	-	1
Patient withdrawn from study	-	1	-	1	-	1
Interview missed ***	1	1	1	1	1	1
Patient too disabled to self report*	-	1	-	1	-	1
Questionnaire missed in interview **	2	1	3	1	1	-
N questionnaires with every item completed +	19	11	17	20	21	18
**Interview 3 (10–12 weeks)**						
Questionnaires returned/Initial patients entered in study arm (%)	26/26 (100%)	23/26 (88%)	26/26 (100%)	22/26 (85%)	26/26 (100%)	23/26 (88%)
**Reasons for non completion**						
Patient died	-	1	-	1	-	1
Patient withdrawn from study	-	1	-	1	-	1
Patient too disabled to self-report*	-	1	-	1	-	1
Questionnaire missed in interview **	-	-	-	1	-	-
N questionnaires with every item completed +	26	19	26	19	24	19

* One patient randomized to standard care was too disabled to complete any self-report questionnaire, at any interview

** The completion of some questionnaires were completed missed at interview with no questions being attempted, although the interview took place and some other questionnaires were completed.

*** One interview was missed because the patient was not available during the interview period and one was missed because of a timing error.

+ Especially early in the study some single items within questionnaires were not completed. In the MSIS, questions with most missing data were 'difficulty carrying things' and 'problems using transport'. In the POS, this was 'feeling good about yourself'. Otherwise there was little pattern in missing data according to specific items.

**Table 4:** Planned and actual times to interview in fast track and standard groups

Time interview planned from baseline	Actual time interval from baseline – mean (SD, median) weeks	
	
	Fast track	Standard
6 weeks	5.54 (1.92, 5)	6.21 (1.71, 5.5)
12 weeks	13.96 (2.93, 13)	12.71 (1.46, 12)
18 weeks (standard only)	N/A	18.73 (2.60, 18)
24–26 weeks (final interview)	25.04 (1.09, 25)	26.33 (3.54, 25)

No significant differences between interview times in the two groups

### Intervention with team

For the 52 patients included in the trial, team actions were: 25 patients, one to three visits leading to assessment that problems were improved and then the patient was discharged; 21 patients, assessment and communication with patient and caregiver, general practitioner and referrer and advice other appropriate action or referral (e.g. to occupational therapy, social services, neurologist etc, note these patients were all assessed as not having specialist palliative care needs according to the referral criteria and so the team felt their main role was to support and refer to other services); and 6 patients received three assessments and were deemed as needing long term follow up and were referral on to community palliative care services. Of the 17 patients excluded from the trial, 3 refused the trial and the team and so received no team care, 1 patient was assessed but did not have MS, 12 received between one and three visits leading to assessment that problems were improved and then the patient was discharged and one patient was cared for and referred on for long-term community palliative care. All the 13 patients cared for by the team were assessed as having specialist palliative care needs, including the 5 'urgent' patients.

### Time taken, location and completeness of data collection at interview

The mean time to complete the baseline (first) interview was 91 minutes (SD = 33); subsequent interviews took on average 61–70 minutes (SD ranged 23–28), and the final interview averaged 82 minutes (SD = 32), overall range 25–150 minutes. In a very few instances the interviewer returned on another day to complete the interview. 87% of interviews occurred in the patient's own home, 6% were in a nursing home and 2% each in hospital, rehabilitation unit, day centre and residential home.

Missing data items were limited. In the baseline interview there was a mean of 5.5, median = 0, items missed per interview (Table 2) out of more than 56 items in the questionnaire. There was one outlier, a patient who had severe cognitive problems, and when this individual's data was excluded the missing data reduced to mean 4.6 items, median 0 items. Over time (except interview 2) the amount of missing questionnaire items reduced to almost none (Table 2). Data for the UNDS and EDSS questionnaire were fully completed, except one interview, apart from the above patient who could not respond to self-report questions. There was some missing data for the MSIS, the POS and the POS-MS. However, this was for particular interviews/patients rather than for specific items. In over 85% of interviews, all items were completed for these questionnaires, and the fast track and standard groups were broadly similar in terms of missing data (Table 3).

Surprisingly there was no consistent relationship between functional disability, measured by EDSS, and missing questionnaire items, supporting our null hypothesis. Significant correlations were not found for interviews at baseline, I3, I4 or I5, and only a weak correlation between higher EDSS score (i.e. greater disability) and missing data for interview 2 (rho= 0.35, p < 0.05). There was, however, a significant relationship between the amount of missing data and the interviewer, see Additional file 1. Interviewer 'B' (who conducted 26 interviews) had on average 10–20 more missing data items per interview than all others. Interviewer 'D' had a slightly higher level of missing data for the baseline interview; all other interviewers had very little missing data. Interviewer A, who had a low rate of missing data, conducted most (137/212, 65%) interviews.

## Discussion

We believe that this paper reports the first use in palliative care of both the MRC Framework and a fast-track controlled trial. Overall we found our methods successful with good compliance, endorsing our use of the MRC Framework and this trial design. Further, we achieved our targeted recruitment within the anticipated timescale, had low attrition (only 5/52 patients) and relatively little missing data. This is a considerable achievement in this difficult area but suggests that it can be reproduced. Because of this patient group, several components of our methods were costly; we conducted mainly home interviews and needed to invest time and resources in the phase I study, although this helped model the service [14,15]. In addition, the existing team caring for patients, including neurologist and MS nurses, were supportive of investigating the potential of palliative care and thus referred appropriate patients in sufficient numbers. This also may have been helped by the phase I study, where other services were involved in modelling the new intervention. This investment paid off, in resulting in a completed, to time, phase II study which achieved its target recruitment.

There are several limitations to this study. We report a phase II study, designed to test the feasibility of the intervention and the evaluation methods. Many phase II studies in the MRC framework are more limited than our trial, often testing the intervention among few patients and not including a randomised component [27]. In contrast, phase II drug therapy trials are often randomised. In our situation, the advantages of a randomised study included decreasing the effects of patient selection bias and the ability to ensure that uniform evaluation criteria were used. Concerned that if the service ran without randomisation, we would never be able to conduct a trial subsequently, and unable to find a suitable non-random comparison group, we appraised the various options for trial. The fast track randomisation appeared to be most feasible. However, at this stage, we were unlikely to have had adequate power for detailed tests comparing the two groups and the broad referral criteria may have resulted in some patients being included who were less likely to benefit from the new service. Despite this, the results we obtained from this study will allow sample size calculation in future studies, and have provided valuable information on the spectrum of needs of people severely affected by MS. As this study is likely to be underpowered, negative or borderline results at this stage should not prevent further development of palliative care for this group of patients.

Different approaches to conducting trials have developed in palliative care. These have included cluster randomisation (where patients in the no service cluster are not aware they are part of a trial) and studies which offered those patients in the control group a 'low level' of service because of fears that patients would not enter the study if they had no service [28]. Many such trials have suffered from selection bias and contamination [29]. In a trial of hospice at home, at the request of the ethics committee patients were randomised 4 (service) to 1 (control), to ensure the service was kept full. In addition, patients referred when the service was 'empty' were offered the service irrespective of their randomisation. This resulted in an underpowered control group that appeared to receive special treatment [30]. All of these studies have proved problematic, with poor recruitment, attrition and bias [9,29].

A fast-track randomised trial is often called a 'wait list' control or delayed intervention trial. They have successfully been used to evaluate cognitive behavioural therapy [31,32], preventive strategies [33], complementary therapies [34] and in rehabilitation [35], neurology and mental health [36]. However to us, and our PAC (which was chaired by and included people affected by MS), the term 'wait list' trial seemed inappropriate. In fact, patients were being offered something much more quickly then they would usually receive it, and so the term 'fast track' as compared to "standard care" was adopted. We referred to the 'standard best practice' group as such, because both our PAC and the ethical review committee wished to make it clear to participants that they would receive all services as best practice currently dictated with no unusual delays or waits. Such a 'fast track' trial is often not feasible in palliative care, because few control patients would live long enough to receive the intervention. This is not the case among patients with longer-term conditions, who are now being considered for referral to palliative care, and where much remains to be understood about the effectiveness and cost effectiveness of different models of palliative care. Further, consideration needs to be given to the length of time the 'standard care' group should wait before receiving the intervention. As for cross over trials, if the intervention is expected to produce effects very quickly then the standard care group might receive the intervention after a shorter wait than in our study. Phase I modelling, as in our study, could help to determine this.

We had to make several improvements to our design in the early stages of the study. Patients with urgent needs were not randomised (but data recorded) to provide a whole picture. Patients refusing the trial should be offered the standard track intervention, not the fast-track, otherwise the position of equipoise is not maintained and patients (and/or their carers) are likely to refuse, in the expectation of receiving the service. This is an important principal to make clear to staff, and it was only after we had begun the study that the effects of getting this wrong became clear to us, and we amended the design. We had good compliance and uptake for the trial, achieving our intended numbers and sometimes having more interviews to do than one interviewer could manage, probably because of; a) an uneven distribution of referrals (ranging up to 6 per week, with an increase after training events) and, b) slightly different interview schedules in two groups, with the SI group having one more interview, so some interviews becoming bunched. Although using the relatively large number of interviewers allowed us to examine missing data across interviews, having 10 interviewers in the project may have introduced unreliability, though we attempted to minimise this with training. It may not have been ideal for patients and carers, who may have wished to have the same interviewer throughout the project. In planning projects in the future resources need to be put in place for potentially fluctuating numbers of referrals.

The management of 'urgent' referrals and those with severe cognitive impairment was another challenge. In this instance independent consultants in palliative medicine spoke to the referrers and attempted to screen out cases where need was not acute. However, it may be that those who were deemed as urgent or very impaired by referrers were also the group whom palliative care would benefit most, reducing the chance of finding a difference in our study. The teams assessment and actions seems to support this: the team assessed as having specialist palliative care needs all 13 (excluding refusals or not MS) patients who were referred directly to the service outside of the trial, but only 31/52 of those randomised. Although the clinical team collected outcome data on these urgent and other referrals, so there is an opportunity to describe the group, this data is likely to be incomplete and may be biased. In the future we would advise that these patients are recruited for data collection by interviewers if possible, so that their circumstances can be independently verified. However, this will require additional resources for interviews.

Compared to many studies in palliative care [37,38] our missing data was slight, and considerably less than the usual levels found in palliative care, where data can be missing in trials and longitudinal studies for up to 50–70% of participants [37-42], often because of death or illness. Cluster randomised trials by Addington-Hall et al and Jordhøy et al were only able to include less than 65% of patients at baseline, with rapid attrition even in the first month [9,37]. We were helped by the careful piloting of our methods, having a dedicated research team, the initial service modelling phase (all of which increase the study costs), and by the fact that people affected by MS have a much longer illness trajectory than do people with far advanced cancer. However, we believe we were also helped by the 'fast track' design, and the knowledge that all patients would be offered the service, with waits for it no longer than those for an outpatient appointment. In our study, recruitment was as expected and only 4 patients died during the course of the trial. Interestingly, three of these were in the standard intervention group – although the causes of death seemed unrelated to the MS.

Another problem in palliative care can be missing data for individual questionnaire items at interview. In a randomised controlled trial of a home palliative care service, McWhinney failed to collect enough usable data over time [43]. In our trial, of those patients who remained alive, we conducted 96% of all possible interviews, and missed a median of 0 items, mean 5.5 items out of over 60 per questionnaire (92% of data items collected). Thus, we had relatively little missing data and were able to conduct interviews for around 1 hour with this group, despite a high level of functional impairment. The amount of missing data at interview was not correlated with the level of functional impairment, or the questionnaires used (supporting, to our surprise, our null hypothesis). However, missing data was related to the interviewer. Many of our people with MS were very disabled, making interviews sometimes more difficult to conduct and potentially distressing for interviewers. This suggests that continued training, careful supervision and monitoring are needed, with feedback to and support of interviewers at an early stage. It also suggests that although we had little missing data, we might have been able to reduce this even further. It indicates that highly skilled quantitative interviewers are needed in palliative care research, because they have to deal with sensitive issues while collecting data within standard questionnaires, often not an easy task. There is often an emphasis on the experience and interviewing skills of qualitative researchers, but some funding agencies suggest that quantitative interviews need not be so experienced. However, our study suggests that skilled and well-trained quantitative interviewers are also important for the quality of research data. This has important implications in some countries: for example in the UK there is a belief that central National Cancer Research Institute nurses, who are involved in a range of cancer studies, could be an interviewing resource for palliative care studies. Our study suggests that careful training and assessment would be needed to establish if this were the case.

## Conclusion and Recommendations

**Figure 3:**
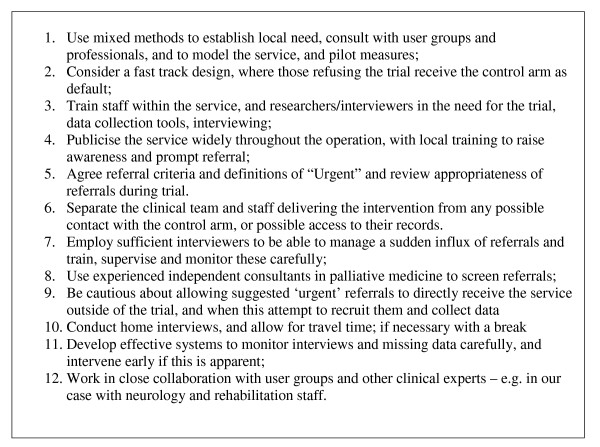
Recommendations for those developing new palliative care services, especially for those patients with longer trajectories.

Our experience suggests that the use of fast track randomised controlled trial (sometimes called a wait list trial) used within the context of the MRC Framework, is feasible and successful in palliative care, when patients have a longer prognosis and are likely to be alive to receive the service after the 'wait' period. We propose that this trial methodology could be used to evaluate new palliative services in cancer or non-cancer patients who have similar illness trajectories. This might apply to other neurological conditions, e.g. Parkinson's disease, and some organ failures, e.g. COPD, and supportive, primary or earlier stage palliative cancer care. Good collaboration with referring services, in identifying appropriate patients is essential. For those with urgent needs data should be obtained and analysed separately. Our method and experience provides a template for future studies in palliative and supportive care. We also found that missing data is mainly interviewer dependent, requiring careful supervision systems. The standard questionnaires used in this study – MSIS, POS, UNDS – appear to work functionally well and have little missing data in this population. Figure 3 shows our main recommendations for those developing and evaluating new palliative care services (especially for patients expected to have longer trajectories). If followed these should significantly improve the quality of palliative care trials, and take advantage of the major opportunity that currently presents itself to conduct trials among non-cancer patients. There is also a need to educate funders of services and research to provide sufficient resources to conduct evaluation of services in this way.

## Competing interests

The authors declare that they have no competing interests.

## Authors' contributions

IJH co-conceived the study and design, co-applied for funding, oversaw all study conduct and developed the analysis plan. PE co-conceived the study and design, led the application for funding and oversaw all conduct of the study. ES co-applied for funding and contributed to the design and study conduct, as well as providing neurological service. TS was an interim research fellow on the project, and helped to refine interview techniques in phase II. RB was appointed as consultant to the service, conducted and analysed interviews for phase I, collected clinical data and oversaw the clinical service activity. SH was appointed as research associate on the project, entered and analysed data in phase II, and contributed to the design of and ethics submission for the post interview survey. SH, TS, IJH and PE all conducted interviews in phase II. IJH led the drafting of this paper (working closely with PE, RB and SH). All other authors saw or contributed to or commented on the final draft. IJH acts as guarantor.

## Pre-publication history

The pre-publication history for this paper can be accessed here:



## Supplementary Material

Additional file 1 Pattern of missing data by interviewer^(b)^ Click here for file
