# Supplementary material for: Randomised controlled trial of a new palliative care service: Compliance, recruitment and completeness of follow-up
Source: BMC Palliat Care. 2008 May 28;7:7. doi: 10.1186/1472-684X-7-7 (PMC2442830; doi:10.1186/1472-684X-7-7)
Supplement: Additional file 1 — Pattern of missing data by interviewer(b) [file 1472-684X-7-7-S1.doc]

| **Interviewer** | **Baseline** | | | | | **I2 (6 weeks)** | | | | | **I3 (12 weeks)** | | | | | **I4 (18 weeks- standard care group only)** | | | | | **Final (24 weeks)** | | | | |
| --- | --- | --- | --- | --- | --- | --- | --- | --- | --- | --- | --- | --- | --- | --- | --- | --- | --- | --- | --- | --- | --- | --- | --- | --- | --- |
|  | *n* | **Mean (#)** | SD (#) | Min (#) | Max (#) | *n* | **Mean (#)** | SD (#) | Min (#) | Max (#) | *n* | **Mean (#)** | SD (#) | Min (#) | Max (#) | *n* | **Mean (#)** | SD (#) | Min (#) | Max (#) | *n* | **Mean (#)** | SD (#) | Min (#) | Max (#) |
| **A** | *15* | **0.1** | 0.3 | 0 | 1 | *27* | **0.1** | 0.3 | 0 | 1 | *37* | **0.1** | 0.4 | 0 | 2 | *16* | **0.0** | 0 | 0 | 0 | *43* | **0.2** | 0.15 | 0 | 1 |
| **B** | *16* | **12.9** | 12.9 | 1 | 36 | *7* | **34.1** | 22.7 | 2 | 56 | *2* | **20.5** | 17.7 | 8 | 33 | *1* | **22.0** | -- | 22 | 22 |  |  |  |  |  |
| **C** | *10* | **0.1** | 0.3 | 0 | 1 | *5* | **0.2** | 0.4 | 0 | 1 | *6* | **0.2** | 0.4 | 0 | 1 |  |  |  |  |  | *1* | **0.0** | -- | 0 | 0 |
| **D** | *4* | **5.8** | 4.9 | 1 | 12 | *2* | **1.0** | 0.0 | 1 | 1 |  |  |  |  |  |  |  |  |  |  |  |  |  |  |  |
| **E** | *1* | **0.0** | -- | 0 | 0 | *2* | **0.5** | 0.7 | 0 | 1 |  |  |  |  |  | *1* | **0.0** | -- | 0 | 0 | *1* | **0.0** | -- | 0 | 0 |
| **F** | *1* | **0.0** | -- | 0 | 0 |  |  |  |  |  | *2* | **0.0** | -- | 0 | 0 | *1* | **2.0** | -- | 2 | 2 | *1* | **0.0** | -- | 0 | 0 |
| **G** |  |  |  |  |  |  |  |  |  |  | *1* | **0.0** | -- | 0 | 0 |  |  |  |  |  |  |  |  |  |  |
| **H** | *1* | **0.0** | -- | 0 | 0 | *2* | **0.0** | -- | 0 | 0 | *1* | **0.0** | -- | 0 | 0 |  |  |  |  |  |  |  |  |  |  |
| **I** | *3* | **0.3** | 0.6 | 0 | 1 | *2* | **0.0** | -- | 0 | 0 |  |  |  |  |  |  |  |  |  |  |  |  |  |  |  |
| **J** |  |  |  |  |  |  |  |  |  |  |  |  |  |  |  | *1* | **0.0** | -- | 0 | 0 |  |  |  |  |  |
| **TOTAL** | *51* | **4.5** | 9.3 | 0 | 36 | *47* | **5.1** | 14.6 | 0 | 56 | *49* | **1.0** | 4.8 | 0 | 33 | *20* | **0.6** | 3.3 | 0 | 22 | *46* | **0.2** | 0.15 | 0 | 0 |

(b) Excluding the one patient who could not complete a questionnaire in any interview, and three missed interviews at I2 (x2) and I4 (x1) because of patients being away / in hospital and one mis-timing for interview

Note this data should be treated with caution because the data is observational, and although several interviewers interviewed the same patients (at different stages in the trial) and in particular in the later interviews one interviewer interviewed nearly all the patients in the study, the allocation of patients to interviewers was not done using a formal random procedure, but was allocated depending on which interviewer was available to interview a patient at a particular time or were interviewing nearby. The one patient who had all items missing and the missed interviews have been excluded as this was not affected by interviewer.

One-way ANOVA, Differences between interviewers was significant at every interview except I5, when there were only two interviewers.

I1: F=4.1, p<0.005, df=7

I2: F=14.8, p<0.001, df=6

I3: F=21.4, p<0.001, df=5

I4 – Not possible to compute as only two interviewers has any missing data at this point

I5 - No difference between interviewers
